# *catena*-Poly[[[aqua­manganese(III)]-μ-(*E*)-5-bromo-*N*-[2-(5-bromo-2-oxidobenzyl­idene­amino)-4-nitro­phen­yl]-2-oxidobenzamidato] *N*,*N*-dimethyl­fomamide monosolvate]

**DOI:** 10.1107/S1600536812008501

**Published:** 2012-03-03

**Authors:** Abeer Mohamed Farag, Teoh Siang Guan, Hasnah Osman, Madhukar Hemamalini, Hoong-Kun Fun

**Affiliations:** a School of Chemical Sciences, Universiti Sains Malaysia, 11800 USM, Penang, Malaysia; b X-ray Crystallography Unit, School of Physics, Universiti Sains Malaysia, 11800 USM, Penang, Malaysia

## Abstract

The asymmetric unit of the title complex, {[Mn(C_20_H_10_Br_2_N_3_O_5_)(H_2_O)]·(CH_3_)_2_NCHO}_*n*_, consists of one Mn^III^ ion, one (*E*)-5-bromo-*N*-[2-(5-bromo-2-oxidobenzyl­idene­amino)-4-nitro­phen­yl]-2-oxidobenzamidate ligand (Schiff base), one water mol­ecule and an *N*,*N*-dimethyl­formamide mol­ecule. The coordination geometry around the Mn^III^ ion is a distorted octa­hedron, being surrounded by two O and two N atoms from the Schiff base, which are positioned in the equatorial plane. The water mol­ecule and the O atom of the carbonyl group from the adjacent Mn^III^ complex are situated at the axial positions, leading to a polymeric chain along the *c* axis. In the crystal, the complex and *N*,*N*-dimethyl­formamide mol­ecules are connected *via* O—H⋯O, C—H⋯O and C—H⋯Br hydrogen bonds, forming a three-dimensional network.

## Related literature

For details of the coordination chemistry and biological importance of manganese, see: Maneiro *et al.* (2003 ▶); Chandra *et al.* (2009 ▶); Chrisianson & Cox (1999 ▶); Ni *et al.* (2009 ▶); Zhang *et al.* (2005 ▶); Huh & Lee (2008 ▶); Pastoriza-Santos & Liz-Marzań (2009 ▶). For related structures, see: Su & Xu (2005 ▶); Ma *et al.* (2004 ▶). For the stability of the temperature controller used in the data collection, see: Cosier & Glazer (1986 ▶).
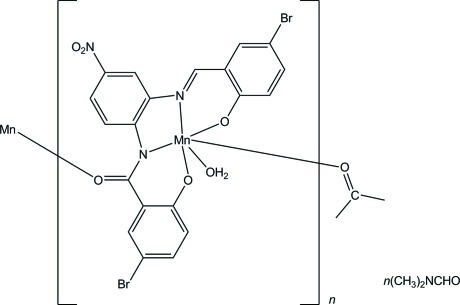


## Experimental

### 

#### Crystal data

[Mn(C_20_H_10_Br_2_N_3_O_5_)(H_2_O)]·C_3_H_7_NO
*M* *_r_* = 678.18
Monoclinic, 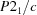

*a* = 11.0746 (6) Å
*b* = 24.9781 (13) Å
*c* = 9.5563 (5) Å
β = 114.658 (1)°
*V* = 2402.4 (2) Å^3^
*Z* = 4
Mo *K*α radiation
μ = 3.93 mm^−1^
*T* = 100 K
0.52 × 0.17 × 0.11 mm

#### Data collection

Bruker APEXII DUO CCD area-detector diffractometer
Absorption correction: multi-scan (*SADABS*; Bruker, 2009 ▶) *T*_min_ = 0.234, *T*_max_ = 0.672
27005 measured reflections
8188 independent reflections
6617 reflections with *I* > 2σ(*I*)
*R*_int_ = 0.032

#### Refinement

*R*[*F* ^2^ > 2σ(*F* ^2^)] = 0.034
*wR*(*F* ^2^) = 0.085
*S* = 1.02
8188 reflections
344 parameters
H atoms treated by a mixture of independent and constrained refinement
Δρ_max_ = 1.85 e Å^−3^
Δρ_min_ = −0.62 e Å^−3^

### 

Data collection: *APEX2* (Bruker, 2009 ▶); cell refinement: *SAINT* (Bruker, 2009 ▶); data reduction: *SAINT*; program(s) used to solve structure: *SHELXTL* (Sheldrick, 2008 ▶); program(s) used to refine structure: *SHELXTL*; molecular graphics: *SHELXTL*; software used to prepare material for publication: *SHELXTL* and *PLATON* (Spek, 2009 ▶).

## Supplementary Material

Crystal structure: contains datablock(s) global, I, an. DOI: 10.1107/S1600536812008501/is5080sup1.cif

Structure factors: contains datablock(s) I. DOI: 10.1107/S1600536812008501/is5080Isup2.hkl

Additional supplementary materials:  crystallographic information; 3D view; checkCIF report

## Figures and Tables

**Table 1:** Hydrogen-bond geometry (Å, °)

*D*—H⋯*A*	*D*—H	H⋯*A*	*D*⋯*A*	*D*—H⋯*A*
O1*W*—H1*W*2⋯O5^i^	0.73 (4)	2.03 (4)	2.736 (2)	163 (4)
O1*W*—H2*W*2⋯O1^ii^	0.77 (3)	2.55 (3)	3.178 (2)	140 (3)
O1*W*—H2*W*2⋯O2^ii^	0.77 (3)	2.19 (3)	2.890 (2)	153 (3)
C2—H2⋯O5^iii^	0.93	2.42	3.351 (2)	175
C5—H5⋯O4^iv^	0.93	2.49	3.395 (3)	166
C7—H7⋯O3^iv^	0.93	2.60	3.509 (2)	167
C18—H18⋯Br2^v^	0.93	2.83	3.449 (2)	125
C23—H23*A*⋯O5^vi^	0.96	2.40	3.350 (3)	170

Symmetry codes: (i) 
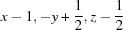
; (ii) 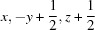; (iii) 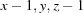; (iv) 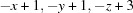; (v) 
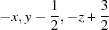
; (vi) 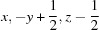.

**Fig. 1.**
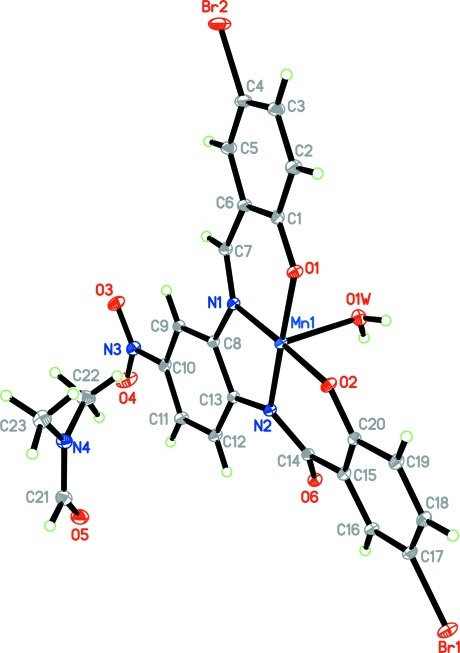
The asymmetric unit of the title compound, showing 30% probability displacement ellipsoids and the atom-numbering scheme.

**Fig. 2.**
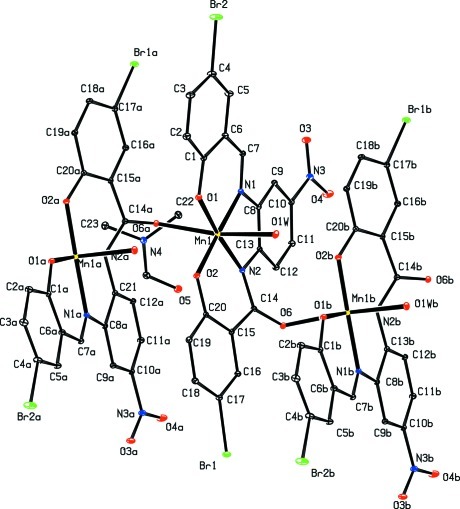
: The polymeric chain of the title compound along the c-axis. H atoms and solvent molecules omitted for clarity.

**Fig. 3.**
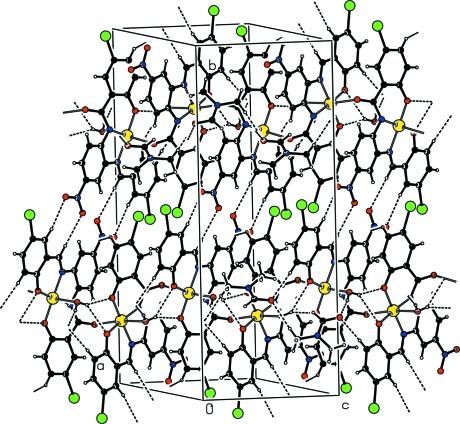
The crystal packing of the title compound, showing a 3D molecular network.

## supplementary crystallographic information

### Comment

In recent years, the coordination chemistry of manganese has been intensively studied due to its presence in the active sites of some enzymes that participate in the chemistry of reactive oxygen species, such as the participation of Mn(II) complexes in peroxidase activity (Maneiro *et al.*, 2003), antipathogenic activity (Chandra *et al.*, 2009) and as essential cofactors in metalloenzymes (Chrisianson & Cox, 1999). X-Ray crystallography has shown a quasi- two-coordinate strongly bent geometry with Mn, which has an almost linear geometry with a wide N—Mn—N angle of 176.1° (Ni *et al.*, 2009). Octahedral coordination is completed by N_4_O_2_ ligands (Zhang *et al.*, 2005). The reaction system (solvent composition and reaction temperature) may cause dramatic changes in the structures. The preparation of the polymer results in the introduction of guest molecules such as DMF into its empty channels by the use of mixed solvents DMF-EtOH-H_2_O under hydro(solvo)thermal conditions (Huh & Lee, 2008). The mechanism of oxidation by DMF have been proposed by Pastoriza-Santos & Liz-Marzań (2009). Due to these interesting features, the title compound was synthesized and its crystal structure presented here.

The asymmetric unit of the title polymeric complex, consisting of one Mn (III) ion, one (*E*)-5-bromo-*N*-(2- (5-bromo-2-hydroxybenzylideneamino)-4-nitrophenyl)-2-hydroxybenzamide (Schiff base), one water molecule and a dimethylformamide solvate molecule is shown in Fig. 1. The coordination geometry around Mn (III) is a distorted octahedron, with the Mn (III) ion being surrounded by two O and two N atoms from the Schiff base which are positioned in the equatorial plane. The water molecule and an atom from a carbonyl group are situated in the axial positions. The carbonyl groups bridge the Mn (III) ions, leading to polymeric chains along the *c*-axis (Fig. 2). The bond lengths are Mn1—O2 = 1.8557 (13); Mn1—O1 = 1.8875 (13); Mn1—N2 = 1.9750 (15); Mn1—N1 = 1.9782 (15); Mn1—O1W =2.2431(15; Mn1—O6 = 2.3448 (14) Å. All bond lengths are in agreement with those in the related structures (Su & Xu, 2005; Ma *et al.*, 2004). In the crystal, (Fig. 3), the complexes are connected with the solvent molecules *via* O—H···O, C—H···O and C—H···Br hydrogen bonds (Table 1) to form a three-dimensional network.

### Experimental

To the solution of 4-nitrobenzene-1,2-diamine (0.306 g, 2 mmol) in ethanol (30 mL) was added 5-bromo-2-hydroxybenzaldehyde (0.804 g, 4 mmol), after which the colour of solution became orange. The mixture was refluxed with stirring for three hours. On adding manganese(II) chloride (0.395 g, 2 mmol), followed by triethylamine (500 mL, 3.6 mmol), a brown precipitate was formed. The mixture was stirred with reflux for three hours. The precipitate, obtained by filtration, was washed by ethanol (5 mL) and dried, affording the title compound (87.33 % yield). Brown block-shaped single crystals suitable for X-ray structure determination were obtained from DMF: ethanol mixture (2:8) with decomposition pt. >340 °C. IR spectroscopy (KBr): ν = 3370 (-OH), 1612 (C═N), 1335 (NO_2_), 639 (M–N), 480 (M–O) cm^-1^. Anal. Calcd (found) for [C_20_H_15_Br_2_N_3_O_6_Mn].C_3_H_7_NO: C 39.50 (39.55), H 2.49 (2.11), N 6.91 (6.55), Mn 9.03 (8.66). MASS Calcd (found) m/z: [605.87 - 2H_2_O]^+^ = 569.87 (571.9).

### Refinement

Atoms H1W2 and H2W2 were located from a difference Fourier map and refined freely [O—H = 0.72 (4) and 0.77 (3) Å]. The remaining H atoms were positioned geometrically [C—H = 0.93–0.96 Å] and were refined using a riding model, with *U*_iso_(H) = 1.2 or 1.5 *U*_eq_(C). A rotating group model was applied to the methyl groups. The highest residual electron density peak is located at 0.75 Å from atom Br1 and the deepest hole is located at 0.66 Å from atom Br2.

### Crystal data

**Table:**

[Mn(C_20_H_10_Br_2_N_3_O_5_)(H_2_O)]·C_3_H_7_NO	*F*(000) = 1344
*M**_r_* = 678.18	*D*_x_ = 1.875 Mg m^−^^3^
Monoclinic, *P*2_1_/*c*	Mo *K*α radiation, λ = 0.71073 Å
Hall symbol: -P 2ybc	Cell parameters from 7902 reflections
*a* = 11.0746 (6) Å	θ = 3.2–31.8°
*b* = 24.9781 (13) Å	µ = 3.93 mm^−^^1^
*c* = 9.5563 (5) Å	*T* = 100 K
β = 114.658 (1)°	Block, brown
*V* = 2402.4 (2) Å^3^	0.52 × 0.17 × 0.11 mm
*Z* = 4	

### Data collection

**Table:**

Bruker APEXII DUO CCD area-detector diffractometer	8188 independent reflections
Radiation source: fine-focus sealed tube	6617 reflections with *I* > 2σ(*I*)
Graphite monochromator	*R*_int_ = 0.032
φ and ω scans	θ_max_ = 31.8°, θ_min_ = 2.6°
Absorption correction: multi-scan (*SADABS*; Bruker, 2009)	*h* = −16→16
*T*_min_ = 0.234, *T*_max_ = 0.672	*k* = −36→37
27005 measured reflections	*l* = −14→14

### Refinement

**Table:**

Refinement on *F*^2^	Primary atom site location: structure-invariant direct methods
Least-squares matrix: full	Secondary atom site location: difference Fourier map
*R*[*F*^2^ > 2σ(*F*^2^)] = 0.034	Hydrogen site location: inferred from neighbouring sites
*wR*(*F*^2^) = 0.085	H atoms treated by a mixture of independent and constrained refinement
*S* = 1.02	*w* = 1/[σ^2^(*F*_o_^2^) + (0.041*P*)^2^ + 1.7977*P*] where *P* = (*F*_o_^2^ + 2*F*_c_^2^)/3
8188 reflections	(Δ/σ)_max_ = 0.003
344 parameters	Δρ_max_ = 1.85 e Å^−^^3^
0 restraints	Δρ_min_ = −0.62 e Å^−^^3^

### Special details

**Table:**

Experimental. The crystal was placed in the cold stream of an Oxford Cryosystems Cobra open-flow nitrogen cryostat (Cosier & Glazer, 1986) operating at 100.0 (1)K.
Geometry. All s.u.'s (except the s.u. in the dihedral angle between two l.s. planes) are estimated using the full covariance matrix. The cell s.u.'s are taken into account individually in the estimation of s.u.'s in distances, angles and torsion angles; correlations between s.u.'s in cell parameters are only used when they are defined by crystal symmetry. An approximate (isotropic) treatment of cell s.u.'s is used for estimating s.u.'s involving l.s. planes.
Refinement. Refinement of F^2^ against ALL reflections. The weighted R-factor wR and goodness of fit S are based on F^2^, conventional R-factors R are based on F, with F set to zero for negative F^2^. The threshold expression of F^2^ > 2σ(F^2^) is used only for calculating R-factors(gt) etc. and is not relevant to the choice of reflections for refinement. R-factors based on F^2^ are statistically about twice as large as those based on F, and R- factors based on ALL data will be even larger.

### Fractional atomic coordinates and isotropic or equivalent isotropic displacement parameters (Å^2^)

**Table:**

	*x*	*y*	*z*	*U*_iso_*/*U*_eq_	
Mn1	0.32207 (3)	0.285186 (10)	1.10714 (3)	0.01283 (6)	
Br1	0.39872 (2)	−0.002567 (8)	1.29527 (2)	0.02540 (6)	
Br2	0.00337 (3)	0.540295 (9)	0.75613 (3)	0.03831 (8)	
O1	0.19454 (14)	0.31410 (5)	0.92195 (14)	0.0161 (2)	
O2	0.28189 (14)	0.21775 (5)	1.01810 (15)	0.0162 (3)	
O3	0.65113 (16)	0.47920 (6)	1.64497 (17)	0.0250 (3)	
O4	0.76182 (19)	0.42584 (7)	1.83364 (18)	0.0345 (4)	
N1	0.37015 (15)	0.35574 (6)	1.20959 (17)	0.0132 (3)	
N2	0.44814 (15)	0.25853 (6)	1.31031 (17)	0.0130 (3)	
N3	0.68440 (17)	0.43404 (7)	1.69974 (19)	0.0196 (3)	
C1	0.15509 (18)	0.36422 (7)	0.8920 (2)	0.0155 (3)	
C2	0.0566 (2)	0.37633 (8)	0.7440 (2)	0.0207 (4)	
H2	0.0211	0.3490	0.6722	0.025*	
C3	0.0124 (2)	0.42798 (8)	0.7046 (2)	0.0239 (4)	
H3	−0.0516	0.4355	0.6063	0.029*	
C4	0.0636 (2)	0.46918 (8)	0.8124 (3)	0.0240 (4)	
C5	0.1582 (2)	0.45925 (8)	0.9576 (2)	0.0212 (4)	
H5	0.1906	0.4870	1.0283	0.025*	
C6	0.20647 (19)	0.40668 (7)	0.9997 (2)	0.0163 (3)	
C7	0.30926 (19)	0.40035 (7)	1.1515 (2)	0.0154 (3)	
H7	0.3347	0.4306	1.2137	0.019*	
C8	0.47310 (18)	0.35266 (7)	1.36140 (19)	0.0130 (3)	
C9	0.53061 (18)	0.39672 (7)	1.4535 (2)	0.0149 (3)	
H9	0.5039	0.4313	1.4178	0.018*	
C10	0.62842 (19)	0.38799 (7)	1.5994 (2)	0.0157 (3)	
C11	0.6734 (2)	0.33696 (8)	1.6552 (2)	0.0177 (3)	
H11	0.7412	0.3324	1.7530	0.021*	
C12	0.6157 (2)	0.29309 (7)	1.5628 (2)	0.0169 (3)	
H12	0.6452	0.2588	1.5986	0.020*	
C13	0.51269 (18)	0.30003 (7)	1.4150 (2)	0.0135 (3)	
C14	0.44056 (18)	0.20846 (7)	1.3632 (2)	0.0141 (3)	
C15	0.38604 (18)	0.16400 (7)	1.2496 (2)	0.0142 (3)	
C16	0.41170 (19)	0.11188 (7)	1.3105 (2)	0.0162 (3)	
H16	0.4619	0.1069	1.4155	0.019*	
C17	0.3626 (2)	0.06804 (7)	1.2151 (2)	0.0177 (3)	
C18	0.2833 (2)	0.07434 (7)	1.0590 (2)	0.0182 (3)	
H18	0.2489	0.0446	0.9964	0.022*	
C19	0.25639 (19)	0.12505 (7)	0.9983 (2)	0.0166 (3)	
H19	0.2018	0.1294	0.8943	0.020*	
C20	0.30947 (18)	0.17067 (7)	1.0900 (2)	0.0138 (3)	
O6	0.47681 (14)	0.19817 (5)	1.50376 (15)	0.0162 (3)	
O1W	0.15751 (15)	0.28059 (7)	1.18541 (18)	0.0203 (3)	
O5	0.94801 (16)	0.27762 (6)	1.48344 (18)	0.0259 (3)	
N4	0.84158 (18)	0.32089 (7)	1.2543 (2)	0.0202 (3)	
C21	0.9247 (2)	0.28447 (8)	1.3460 (3)	0.0232 (4)	
H21	0.9687	0.2625	1.3039	0.028*	
C22	0.7663 (2)	0.35587 (9)	1.3091 (3)	0.0269 (4)	
H22A	0.7843	0.3468	1.4135	0.040*	
H22B	0.6731	0.3516	1.2455	0.040*	
H22C	0.7915	0.3924	1.3044	0.040*	
C23	0.8221 (2)	0.32762 (9)	1.0947 (2)	0.0250 (4)	
H23A	0.8686	0.2998	1.0680	0.037*	
H23B	0.8558	0.3619	1.0826	0.037*	
H23C	0.7290	0.3255	1.0285	0.037*	
H1W2	0.110 (4)	0.2602 (15)	1.142 (4)	0.051 (11)*	
H2W2	0.180 (3)	0.2712 (12)	1.269 (4)	0.034 (8)*	

### Atomic displacement parameters (Å^2^)

**Table:**

	*U*^11^	*U*^22^	*U*^33^	*U*^12^	*U*^13^	*U*^23^
Mn1	0.01673 (13)	0.01111 (12)	0.00881 (11)	−0.00105 (9)	0.00349 (10)	−0.00144 (9)
Br1	0.03687 (12)	0.01250 (9)	0.02373 (10)	0.00229 (8)	0.00957 (9)	0.00146 (7)
Br2	0.03147 (13)	0.01713 (10)	0.04152 (14)	0.00359 (8)	−0.00934 (10)	0.00522 (9)
O1	0.0204 (6)	0.0140 (6)	0.0106 (5)	−0.0001 (5)	0.0030 (5)	−0.0002 (4)
O2	0.0241 (7)	0.0107 (6)	0.0119 (5)	−0.0022 (5)	0.0057 (5)	−0.0014 (4)
O3	0.0333 (8)	0.0129 (6)	0.0223 (7)	−0.0034 (6)	0.0050 (6)	−0.0013 (5)
O4	0.0465 (10)	0.0217 (7)	0.0162 (7)	−0.0040 (7)	−0.0059 (7)	−0.0030 (6)
N1	0.0153 (7)	0.0128 (6)	0.0105 (6)	−0.0011 (5)	0.0043 (5)	−0.0012 (5)
N2	0.0158 (7)	0.0113 (6)	0.0106 (6)	−0.0006 (5)	0.0044 (5)	−0.0018 (5)
N3	0.0230 (8)	0.0168 (7)	0.0161 (7)	−0.0034 (6)	0.0052 (6)	−0.0032 (6)
C1	0.0163 (8)	0.0155 (8)	0.0132 (7)	−0.0015 (6)	0.0047 (6)	−0.0003 (6)
C2	0.0209 (9)	0.0195 (9)	0.0151 (8)	−0.0017 (7)	0.0011 (7)	0.0001 (7)
C3	0.0203 (9)	0.0212 (9)	0.0204 (9)	0.0007 (7)	−0.0013 (8)	0.0041 (7)
C4	0.0218 (10)	0.0160 (8)	0.0261 (10)	0.0017 (7)	0.0019 (8)	0.0038 (7)
C5	0.0193 (9)	0.0146 (8)	0.0222 (9)	0.0002 (7)	0.0014 (7)	−0.0002 (7)
C6	0.0172 (8)	0.0150 (8)	0.0137 (8)	−0.0003 (6)	0.0034 (7)	−0.0005 (6)
C7	0.0170 (8)	0.0136 (7)	0.0143 (8)	0.0000 (6)	0.0050 (7)	−0.0012 (6)
C8	0.0138 (8)	0.0128 (7)	0.0107 (7)	−0.0004 (6)	0.0036 (6)	−0.0010 (6)
C9	0.0164 (8)	0.0137 (7)	0.0142 (7)	−0.0011 (6)	0.0059 (6)	−0.0014 (6)
C10	0.0186 (8)	0.0141 (8)	0.0129 (7)	−0.0038 (6)	0.0050 (7)	−0.0043 (6)
C11	0.0195 (9)	0.0167 (8)	0.0125 (7)	−0.0011 (7)	0.0024 (7)	−0.0011 (6)
C12	0.0203 (9)	0.0137 (8)	0.0138 (8)	−0.0010 (7)	0.0044 (7)	−0.0011 (6)
C13	0.0156 (8)	0.0133 (7)	0.0115 (7)	−0.0011 (6)	0.0058 (6)	−0.0019 (6)
C14	0.0150 (8)	0.0129 (7)	0.0134 (7)	−0.0003 (6)	0.0049 (6)	−0.0007 (6)
C15	0.0176 (8)	0.0135 (7)	0.0115 (7)	−0.0009 (6)	0.0059 (6)	−0.0008 (6)
C16	0.0193 (9)	0.0133 (8)	0.0152 (8)	0.0003 (6)	0.0063 (7)	0.0003 (6)
C17	0.0215 (9)	0.0113 (7)	0.0210 (9)	0.0007 (7)	0.0096 (7)	0.0009 (6)
C18	0.0222 (9)	0.0134 (8)	0.0180 (8)	−0.0036 (7)	0.0075 (7)	−0.0040 (6)
C19	0.0205 (9)	0.0150 (8)	0.0126 (7)	−0.0029 (7)	0.0052 (7)	−0.0030 (6)
C20	0.0165 (8)	0.0125 (7)	0.0134 (7)	−0.0007 (6)	0.0073 (6)	−0.0014 (6)
O6	0.0196 (6)	0.0164 (6)	0.0120 (6)	0.0000 (5)	0.0058 (5)	0.0006 (5)
O1W	0.0181 (7)	0.0288 (8)	0.0123 (6)	−0.0024 (6)	0.0046 (5)	0.0010 (6)
O5	0.0249 (8)	0.0265 (8)	0.0253 (7)	0.0055 (6)	0.0093 (6)	0.0059 (6)
N4	0.0217 (8)	0.0207 (8)	0.0197 (8)	0.0021 (6)	0.0099 (7)	0.0001 (6)
C21	0.0229 (10)	0.0222 (9)	0.0262 (10)	0.0029 (8)	0.0120 (8)	0.0009 (8)
C22	0.0294 (11)	0.0281 (10)	0.0262 (10)	0.0088 (9)	0.0146 (9)	0.0024 (8)
C23	0.0290 (11)	0.0268 (10)	0.0200 (9)	0.0002 (8)	0.0110 (8)	0.0002 (8)

### Geometric parameters (Å, º)

**Table:**

Mn1—O2	1.8557 (13)	C9—H9	0.9300
Mn1—O1	1.8875 (13)	C10—C11	1.391 (3)
Mn1—N2	1.9750 (15)	C11—C12	1.385 (3)
Mn1—N1	1.9782 (15)	C11—H11	0.9300
Mn1—O1W	2.2431 (15)	C12—C13	1.408 (3)
Mn1—O6^i^	2.3448 (14)	C12—H12	0.9300
Br1—C17	1.8977 (18)	C14—O6	1.258 (2)
Br2—C4	1.895 (2)	C14—C15	1.493 (2)
O1—C1	1.317 (2)	C15—C16	1.406 (2)
O2—C20	1.331 (2)	C15—C20	1.412 (2)
O3—N3	1.233 (2)	C16—C17	1.383 (3)
O4—N3	1.224 (2)	C16—H16	0.9300
N1—C7	1.301 (2)	C17—C18	1.389 (3)
N1—C8	1.425 (2)	C18—C19	1.373 (3)
N2—C14	1.365 (2)	C18—H18	0.9300
N2—C13	1.410 (2)	C19—C20	1.407 (2)
N3—C10	1.460 (2)	C19—H19	0.9300
C1—C2	1.412 (3)	O6—Mn1^ii^	2.3448 (14)
C1—C6	1.421 (3)	O1W—H1W2	0.72 (4)
C2—C3	1.375 (3)	O1W—H2W2	0.77 (3)
C2—H2	0.9300	O5—C21	1.240 (3)
C3—C4	1.398 (3)	N4—C21	1.331 (3)
C3—H3	0.9300	N4—C22	1.448 (3)
C4—C5	1.368 (3)	N4—C23	1.458 (3)
C5—C6	1.412 (3)	C21—H21	0.9300
C5—H5	0.9300	C22—H22A	0.9600
C6—C7	1.430 (3)	C22—H22B	0.9600
C7—H7	0.9300	C22—H22C	0.9600
C8—C9	1.387 (2)	C23—H23A	0.9600
C8—C13	1.413 (2)	C23—H23B	0.9600
C9—C10	1.380 (3)	C23—H23C	0.9600
			
O2—Mn1—O1	88.57 (6)	C9—C10—N3	118.59 (16)
O2—Mn1—N2	94.63 (6)	C11—C10—N3	118.96 (16)
O1—Mn1—N2	175.06 (6)	C12—C11—C10	118.98 (17)
O2—Mn1—N1	177.72 (6)	C12—C11—H11	120.5
O1—Mn1—N1	93.71 (6)	C10—C11—H11	120.5
N2—Mn1—N1	83.10 (6)	C11—C12—C13	120.50 (17)
O2—Mn1—O1W	91.93 (6)	C11—C12—H12	119.8
O1—Mn1—O1W	86.45 (6)	C13—C12—H12	119.8
N2—Mn1—O1W	89.68 (6)	C12—C13—N2	125.44 (16)
N1—Mn1—O1W	88.22 (6)	C12—C13—C8	118.51 (16)
O2—Mn1—O6^i^	92.60 (5)	N2—C13—C8	115.98 (15)
O1—Mn1—O6^i^	85.96 (5)	O6—C14—N2	122.76 (16)
N2—Mn1—O6^i^	97.63 (6)	O6—C14—C15	118.47 (16)
N1—Mn1—O6^i^	87.56 (6)	N2—C14—C15	118.77 (15)
O1W—Mn1—O6^i^	171.06 (5)	C16—C15—C20	118.88 (16)
C1—O1—Mn1	128.16 (12)	C16—C15—C14	115.91 (15)
C20—O2—Mn1	127.29 (11)	C20—C15—C14	125.18 (16)
C7—N1—C8	122.21 (15)	C17—C16—C15	120.29 (17)
C7—N1—Mn1	124.56 (13)	C17—C16—H16	119.9
C8—N1—Mn1	113.05 (11)	C15—C16—H16	119.9
C14—N2—C13	120.16 (15)	C16—C17—C18	121.12 (17)
C14—N2—Mn1	123.06 (12)	C16—C17—Br1	120.75 (15)
C13—N2—Mn1	113.00 (11)	C18—C17—Br1	118.11 (14)
O4—N3—O3	123.46 (17)	C19—C18—C17	119.14 (17)
O4—N3—C10	118.37 (17)	C19—C18—H18	120.4
O3—N3—C10	118.17 (16)	C17—C18—H18	120.4
O1—C1—C2	117.94 (16)	C18—C19—C20	121.56 (17)
O1—C1—C6	123.76 (16)	C18—C19—H19	119.2
C2—C1—C6	118.30 (17)	C20—C19—H19	119.2
C3—C2—C1	120.85 (18)	O2—C20—C19	116.61 (16)
C3—C2—H2	119.6	O2—C20—C15	124.49 (16)
C1—C2—H2	119.6	C19—C20—C15	118.89 (16)
C2—C3—C4	120.02 (19)	C14—O6—Mn1^ii^	116.75 (12)
C2—C3—H3	120.0	Mn1—O1W—H1W2	110 (3)
C4—C3—H3	120.0	Mn1—O1W—H2W2	114 (2)
C5—C4—C3	121.17 (19)	H1W2—O1W—H2W2	103 (3)
C5—C4—Br2	119.20 (16)	C21—N4—C22	121.24 (18)
C3—C4—Br2	119.63 (16)	C21—N4—C23	121.86 (18)
C4—C5—C6	119.77 (18)	C22—N4—C23	116.90 (17)
C4—C5—H5	120.1	O5—C21—N4	125.0 (2)
C6—C5—H5	120.1	O5—C21—H21	117.5
C5—C6—C1	119.88 (17)	N4—C21—H21	117.5
C5—C6—C7	116.01 (16)	N4—C22—H22A	109.5
C1—C6—C7	124.10 (17)	N4—C22—H22B	109.5
N1—C7—C6	125.38 (17)	H22A—C22—H22B	109.5
N1—C7—H7	117.3	N4—C22—H22C	109.5
C6—C7—H7	117.3	H22A—C22—H22C	109.5
C9—C8—C13	121.17 (16)	H22B—C22—H22C	109.5
C9—C8—N1	124.31 (16)	N4—C23—H23A	109.5
C13—C8—N1	114.51 (15)	N4—C23—H23B	109.5
C10—C9—C8	118.35 (17)	H23A—C23—H23B	109.5
C10—C9—H9	120.8	N4—C23—H23C	109.5
C8—C9—H9	120.8	H23A—C23—H23C	109.5
C9—C10—C11	122.43 (16)	H23B—C23—H23C	109.5
			
O2—Mn1—O1—C1	175.97 (16)	N1—C8—C9—C10	179.15 (17)
N1—Mn1—O1—C1	−4.03 (16)	C8—C9—C10—C11	1.8 (3)
O1W—Mn1—O1—C1	83.95 (16)	C8—C9—C10—N3	−176.83 (16)
O6^i^—Mn1—O1—C1	−91.32 (15)	O4—N3—C10—C9	173.19 (19)
O1—Mn1—O2—C20	−162.27 (15)	O3—N3—C10—C9	−6.8 (3)
N2—Mn1—O2—C20	13.96 (16)	O4—N3—C10—C11	−5.5 (3)
O1W—Mn1—O2—C20	−75.87 (15)	O3—N3—C10—C11	174.50 (18)
O6^i^—Mn1—O2—C20	111.84 (15)	C9—C10—C11—C12	−1.8 (3)
O1—Mn1—N1—C7	6.45 (16)	N3—C10—C11—C12	176.82 (17)
N2—Mn1—N1—C7	−169.77 (16)	C10—C11—C12—C13	−0.3 (3)
O1W—Mn1—N1—C7	−79.88 (15)	C11—C12—C13—N2	178.99 (18)
O6^i^—Mn1—N1—C7	92.24 (15)	C11—C12—C13—C8	2.2 (3)
O1—Mn1—N1—C8	−178.30 (12)	C14—N2—C13—C12	27.2 (3)
N2—Mn1—N1—C8	5.48 (12)	Mn1—N2—C13—C12	−174.01 (15)
O1W—Mn1—N1—C8	95.38 (12)	C14—N2—C13—C8	−155.88 (16)
O6^i^—Mn1—N1—C8	−92.51 (12)	Mn1—N2—C13—C8	2.9 (2)
O2—Mn1—N2—C14	−26.70 (15)	C9—C8—C13—C12	−2.2 (3)
N1—Mn1—N2—C14	153.46 (15)	N1—C8—C13—C12	178.80 (16)
O1W—Mn1—N2—C14	65.22 (14)	C9—C8—C13—N2	−179.31 (16)
O6^i^—Mn1—N2—C14	−119.95 (14)	N1—C8—C13—N2	1.7 (2)
O2—Mn1—N2—C13	175.27 (12)	C13—N2—C14—O6	7.6 (3)
N1—Mn1—N2—C13	−4.57 (12)	Mn1—N2—C14—O6	−148.89 (15)
O1W—Mn1—N2—C13	−92.82 (13)	C13—N2—C14—C15	−172.84 (16)
O6^i^—Mn1—N2—C13	82.02 (12)	Mn1—N2—C14—C15	30.6 (2)
Mn1—O1—C1—C2	−179.45 (14)	O6—C14—C15—C16	−16.2 (3)
Mn1—O1—C1—C6	0.9 (3)	N2—C14—C15—C16	164.22 (16)
O1—C1—C2—C3	−179.07 (19)	O6—C14—C15—C20	162.02 (18)
C6—C1—C2—C3	0.6 (3)	N2—C14—C15—C20	−17.5 (3)
C1—C2—C3—C4	−0.9 (3)	C20—C15—C16—C17	0.1 (3)
C2—C3—C4—C5	0.3 (4)	C14—C15—C16—C17	178.44 (17)
C2—C3—C4—Br2	179.33 (17)	C15—C16—C17—C18	−2.4 (3)
C3—C4—C5—C6	0.7 (3)	C15—C16—C17—Br1	179.05 (14)
Br2—C4—C5—C6	−178.36 (16)	C16—C17—C18—C19	1.7 (3)
C4—C5—C6—C1	−1.0 (3)	Br1—C17—C18—C19	−179.70 (15)
C4—C5—C6—C7	177.4 (2)	C17—C18—C19—C20	1.3 (3)
O1—C1—C6—C5	−179.99 (18)	Mn1—O2—C20—C19	173.51 (13)
C2—C1—C6—C5	0.4 (3)	Mn1—O2—C20—C15	−5.8 (3)
O1—C1—C6—C7	1.8 (3)	C18—C19—C20—O2	177.07 (17)
C2—C1—C6—C7	−177.89 (18)	C18—C19—C20—C15	−3.6 (3)
C8—N1—C7—C6	179.15 (17)	C16—C15—C20—O2	−177.88 (17)
Mn1—N1—C7—C6	−6.0 (3)	C14—C15—C20—O2	3.9 (3)
C5—C6—C7—N1	−177.16 (19)	C16—C15—C20—C19	2.8 (3)
C1—C6—C7—N1	1.2 (3)	C14—C15—C20—C19	−175.36 (17)
C7—N1—C8—C9	−9.0 (3)	N2—C14—O6—Mn1^ii^	83.77 (19)
Mn1—N1—C8—C9	175.61 (14)	C15—C14—O6—Mn1^ii^	−95.75 (17)
C7—N1—C8—C13	169.94 (17)	C22—N4—C21—O5	−1.6 (3)
Mn1—N1—C8—C13	−5.44 (19)	C23—N4—C21—O5	178.2 (2)
C13—C8—C9—C10	0.3 (3)		

Symmetry codes: (i) *x*, −*y*+1/2, *z*−1/2; (ii) *x*, −*y*+1/2, *z*+1/2.

### Hydrogen-bond geometry (Å, º)

**Table:**

*D*—H···*A*	*D*—H	H···*A*	*D*···*A*	*D*—H···*A*
O1*W*—H1*W*2···O5^iii^	0.73 (4)	2.03 (4)	2.736 (2)	163 (4)
O1*W*—H2*W*2···O1^ii^	0.77 (3)	2.55 (3)	3.178 (2)	140 (3)
O1*W*—H2*W*2···O2^ii^	0.77 (3)	2.19 (3)	2.890 (2)	153 (3)
C2—H2···O5^iv^	0.93	2.42	3.351 (2)	175
C5—H5···O4^v^	0.93	2.49	3.395 (3)	166
C7—H7···O3^v^	0.93	2.60	3.509 (2)	167
C18—H18···Br2^vi^	0.93	2.83	3.449 (2)	125
C23—H23*A*···O5^i^	0.96	2.40	3.350 (3)	170

Symmetry codes: (i) *x*, −*y*+1/2, *z*−1/2; (ii) *x*, −*y*+1/2, *z*+1/2; (iii) *x*−1, −*y*+1/2, *z*−1/2; (iv) *x*−1, *y*, *z*−1; (v) −*x*+1, −*y*+1, −*z*+3; (vi) −*x*, *y*−1/2, −*z*+3/2.
